# Supplementary material for: Dnmt3a2 expression during embryonic development is required for phenotypic stability
Source: Commun Biol. 2025 Dec 8;9:44. doi: 10.1038/s42003-025-09311-1 (PMC12789621; doi:10.1038/s42003-025-09311-1)
Supplement: Supplementary file 2 — Supplementary information [file 42003_2025_9311_MOESM2_ESM.pdf]

Supplementary information

Dnmt3a2 expression during embryonic development is required for phenotypic stability

Minmin Liu[1#], Guillermo Urrutia[1#], Rachel Shereda[1], Galen Hostetter[2], Stacey L. Thomas[1], Gangning Liang[3], Peter A. Jones[1*]

[1]Department of Epigenetics, Van Andel Institute, Grand Rapids, MI. USA.

[2]Pathology and Biorepository Core, Van Andel Institute, Grand Rapids, MI. USA.

[3]Department of Urology, Keck School of Medicine, University of Southern California, Los Angeles, CA. USA

*Correspondence: Peter.Jones@vai.org

[#]These authors contributed equally

```
N-terminal fragment in Dnmt3a1 KO : MPSSGPGDTSSSSLEREDDRKEGEEQEENRGKEERQEPSATARKVGRPGRKRKHPPVESS    60
                          Dnmt3a1 : MPSSGPGDTSSSSLEREDDRKEGEEQEENRGKEERQEPSATARKVGRPGRKRKHPPVESS    60
                                    ************************************************************

N-terminal fragment in Dnmt3a1 KO : DTPKDPAVTTKSQPMAQDSGPSDLLPNGDLEKRSEPQPEEGSPAAGQKGGAPAEGEGTET    120
                          Dnmt3a1 : DTPKDPAVTTKSQPMAQDSGPSDLLPNGDLEKRSEPQPEEGSPAAGQKGGAPAEGEGTET    120
                                    ************************************************************

                                                  Frame shift   Premature termination
N-terminal fragment in Dnmt3a1 KO : PPEASRAVENGCCVTKEGRGASAGEG*---------------------------------    146
                          Dnmt3a1 : PPEASRAVENGCCVTKEGRGASAGEGKEQKQTNIESMKMEGSRGRLRGGLGWESSLRQRP    180
                                    **************************

N-terminal fragment in Dnmt3a1 KO : ------------------------------------------------------------    146
                          Dnmt3a1 : MPRLTFQAGDPYYISKRKRDEWLARWKREAEKKAKVIAVMNAVEENQASGESQKVEEASP    240

N-terminal fragment in Dnmt3a1 KO : ------------------------------------------------------------    146
                          Dnmt3a1 : PAVQQPTDPASPTVATTPEPVGGDAGDKNATKAADDEPEYEDGRGFGIGELVWGKLRGFS    300

                                                       ......

N-terminal fragment in Dnmt3a1 KO : ------------------------------------------------------------    146
                          Dnmt3a1 : DQHFPVFMNEKEDILWCTEMERVFGFPVHYTDVSNMSRLARQRLLGRSWSVPVIRHLFAP    900

N-terminal fragment in Dnmt3a1 KO : ---------        146
                          Dnmt3a1 : LKEYFACV*        908
```

Supplementary Fig. 1: Alignment of the protein sequence of residual N-terminal fragment potentially left in cells after exon 5 and 6 deletion to that of Dnmt3a1. The reading frame shifts after Glycine 146 followed by the premature stop codon.

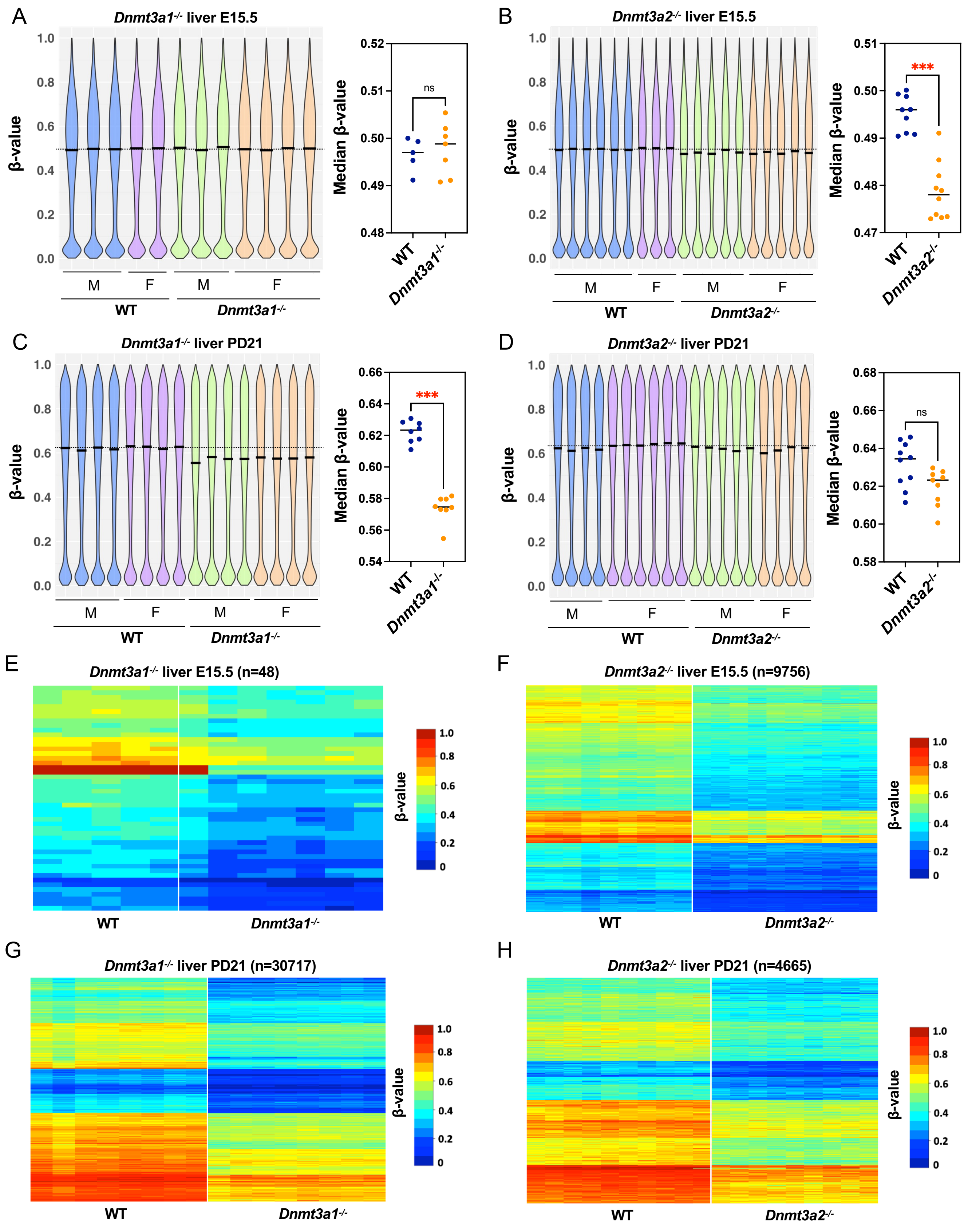
A
Dnmt3a1-/- liver E15.5
β-value
Median β-value
ns
M
F
WT
Dnmt3a1-/-
B
Dnmt3a2-/- liver E15.5
***
Dnmt3a2-/-
C
Dnmt3a1-/- liver PD21
D
Dnmt3a2-/- liver PD21
E
Dnmt3a1-/- liver E15.5 (n=48)
F
Dnmt3a2-/- liver E15.5 (n=9756)
G
Dnmt3a1-/- liver PD21 (n=30717)
H
Dnmt3a2-/- liver PD21 (n=4665)

Supplementary Fig. 2: Hypomethylated CpGs in liver from *Dnmt3a1*$^{-/-}$ and *Dnmt3a2*$^{-/-}$ mice during embryonic and postnatal development. A-D, Distribution of CpG methylation for the autosomal probes from the MM285 array in liver of the *Dnmt3a1*$^{-/-}$ (**A, C**) and *Dnmt3a2*$^{-/-}$ (**B, D**) mice at E15.5 and PD21. The left panels show violin plots for the distribution of CpG methylation as β-values in WT and KO liver. Males are represented as M and females are represented as F. The median β-value in WT liver is marked by the dotted line. The right panels compare the median β-value for the WT to the KO liver. Biological replicates are represented by individual dots. ns, not significant and ***, $p < 0.001$ by two-tailed unpaired Mann-Whitney U test. **E-H,** Heatmap representing the hypomethylated CpGs in liver of the *Dnmt3a1*$^{-/-}$ (**E, G**) and *Dnmt3a2*$^{-/-}$ (**F, H**) mice at E15.5 and PD21. Hypomethylated probes were selected using a cutoff of beta-value difference greater than 0.1 and $p < 0.05$ by two-tailed unpaired Mann-Whitney U test. Methylation levels are represented by a cold to warm color scale (β-value 0-1, 0 – 100% methylated), where every row represents an individual probe, and every column represents sample from an individual mouse.

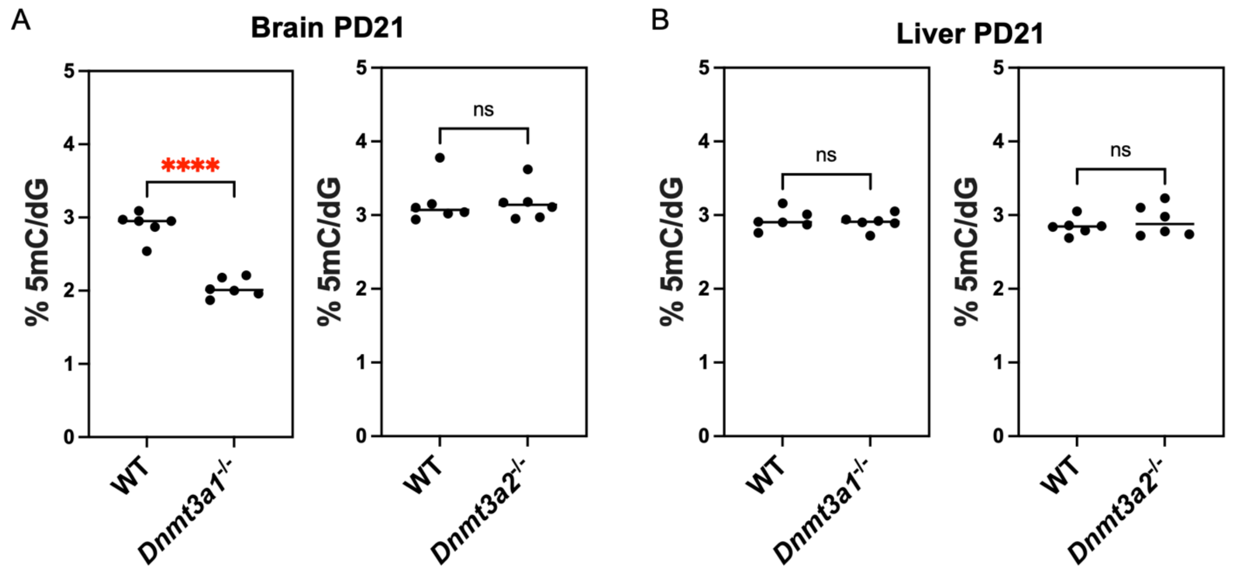

Supplementary Fig. 3: Global DNA methylation levels of brain (A) and liver (B) of the *Dnmt3a1*$^{-/-}$ and *Dnmt3a2*$^{-/-}$ mice at PD21 measured by Liquid Chromatography-Mass Spectrometry (LCMS). Data are represented as the percentage of 5mC to dG as individual dots. **** $p < 0.0001$ (two-sided unpaired t tests).

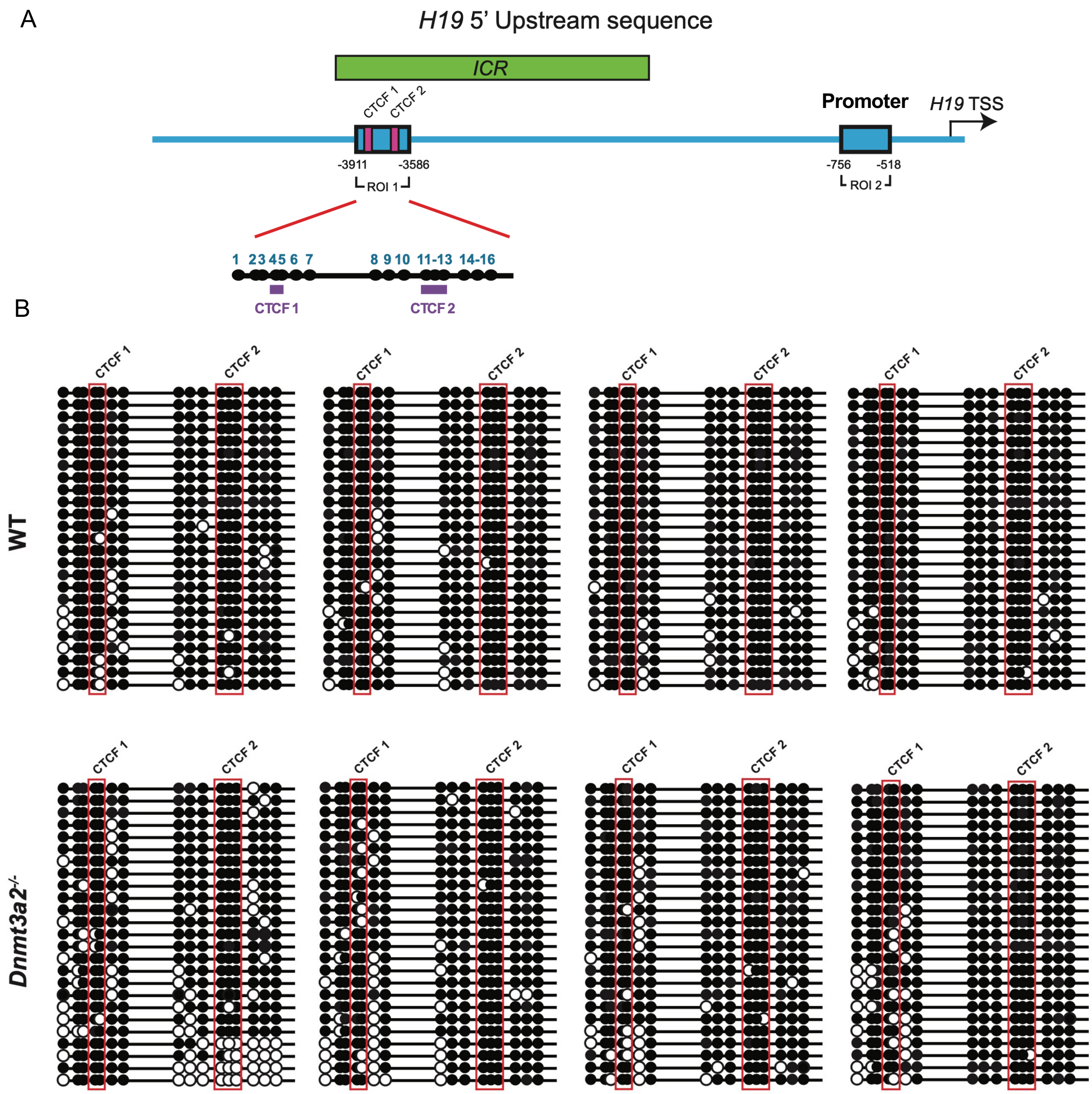

Supplementary Fig. 4: Dnmt3a2 loss induces CTCF binding site loss of methylation at the *H19* ICR. A. Schematic representation of the *H19* 5' Upstream region. The studied region of interest (ROI 1, blue box) is located inside the ICR (Green box) and encompassed two CTCF binding sites (magenta boxes). A 16 CpG-containing 422 bp amplicon was amplified by targeted amplicon bisulfite sequencing to analyze the CpG dinucleotide (black bubbles) methylation. CTCF binding sites are represented underneath CpG dinucleotides as purple boxes. **B.** Bubble plots depicting the methylation status for ROI 1 in individual DNA strands from WT and *Dnmt3a2*$^{-/-}$ mice. 25 unique and randomly selected sequences per animal are displayed (black = methylated CpG, white = unmethylated CpG). Red boxes indicate the *H19* CTCF binding sites.

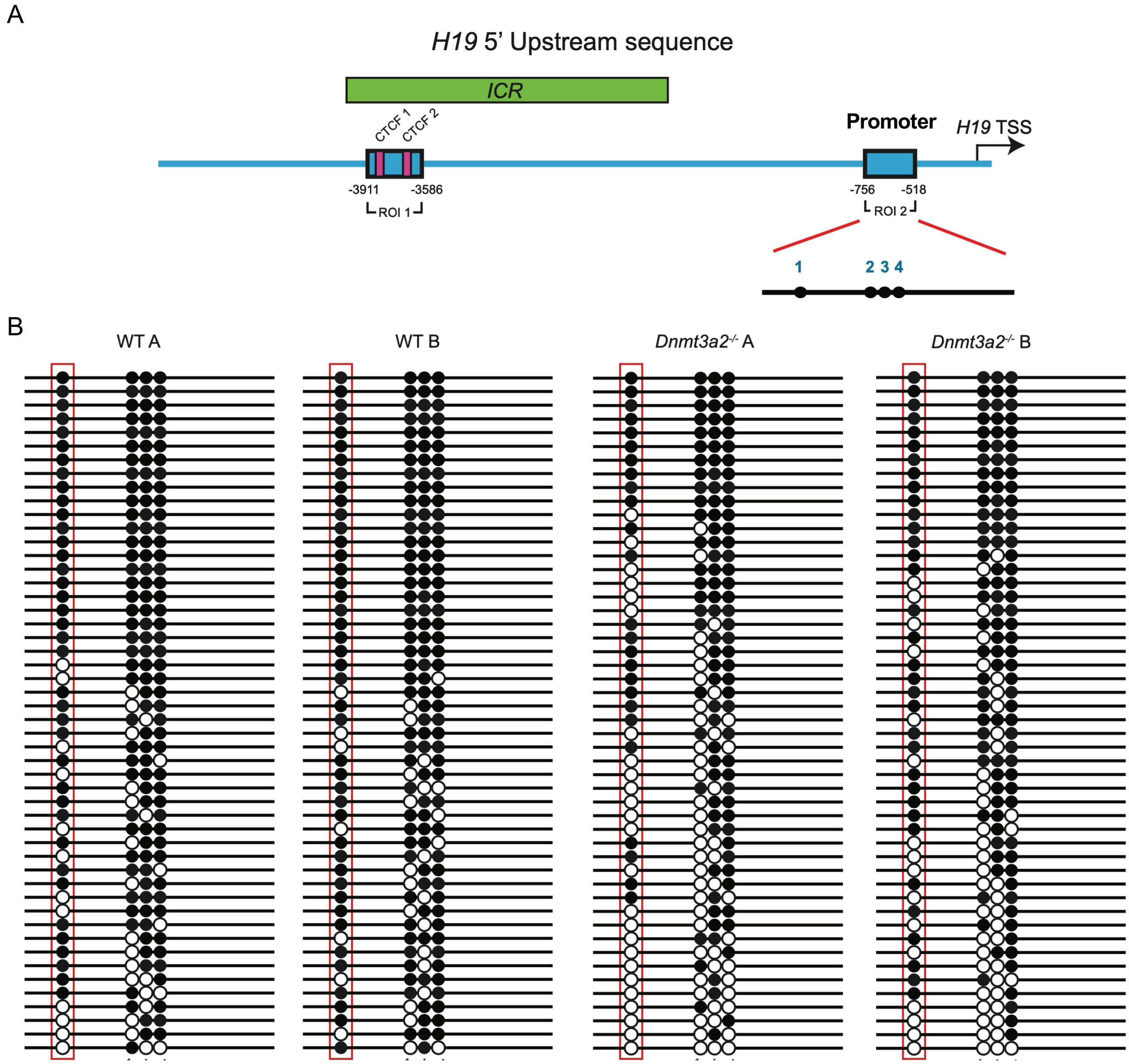

Supplementary Fig. 5: Dnmt3a2 loss induces loss of methylation at the *H19* promoter. A. Schematic representation of the *H19* 5' Upstream region. The studied region of interest (ROI 2, blue box) is located downstream of the ICR (Green box). A 4 CpG-containing 227 bp amplicon was amplified by targeted amplicon bisulfite sequencing to analyze the CpG dinucleotide (black bubbles) methylation. **B.** Bubble plots depicting the methylation status for ROI 2 in individual DNA strands from WT and *Dnmt3a2*$^{-/-}$ mice. 50 unique and randomly selected sequences per animal are displayed (black = methylated CpG, white = unmethylated CpG). Red box indicates the CpG site detected by the EPIC array probe.

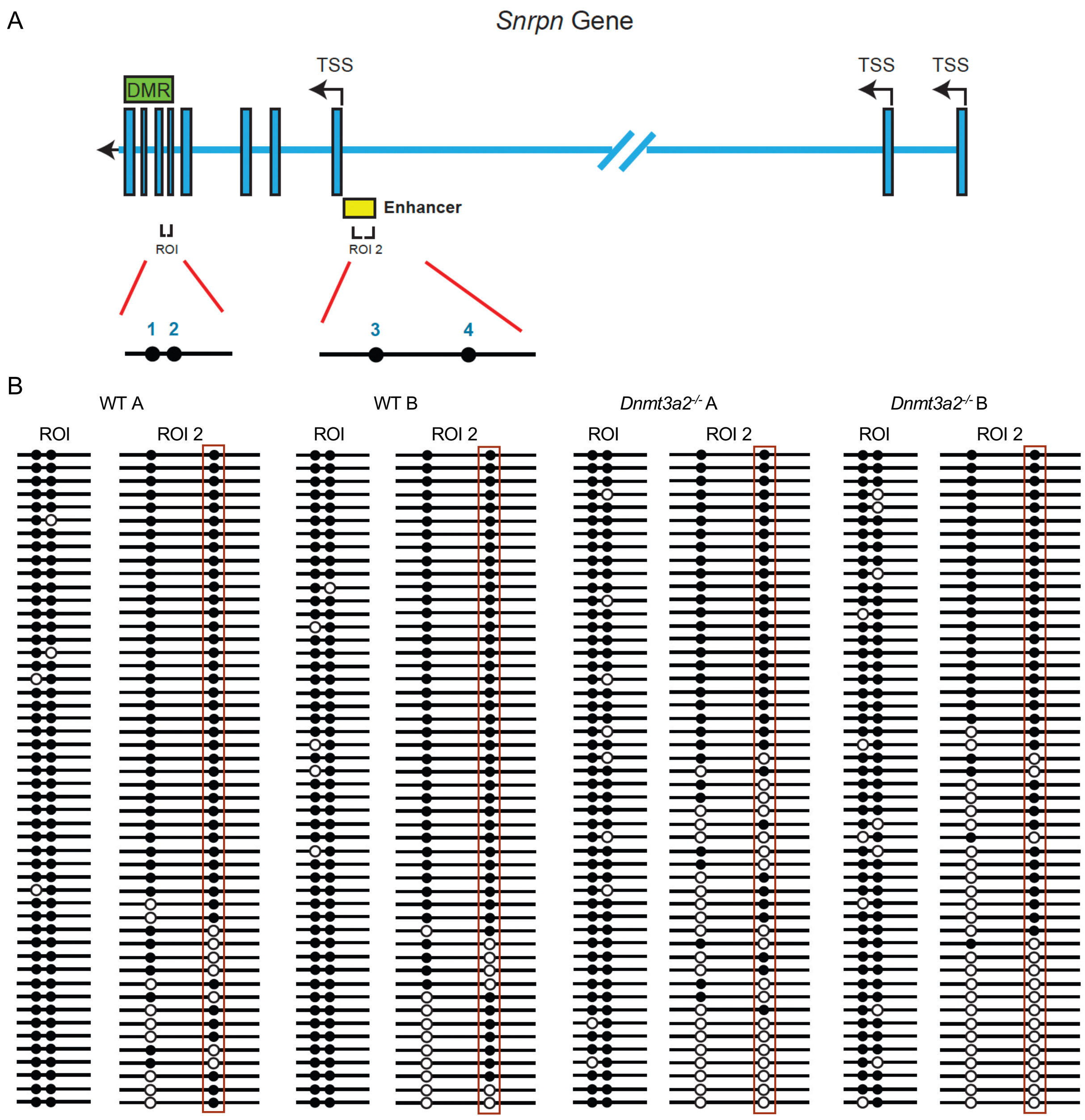

Supplementary Fig. 6: Dnmt3a2 loss induces stochastic loss of methylation at the *Snrpn* gene. A. Schematic representation of the *Snrpn* gene (exons are depicted as blue boxes). The DMR is represented by a green box and the enhancer by a yellow box. The regions of interest, namely ROI (134 bp) and ROI 2 (259 bp) were amplified by targeted amplicon bisulfite sequencing to analyze the CpG dinucleotide (black bubbles) methylation. **B.** Bubble plots depicting the methylation status for *Snrpn* ROI and ROI 2 in individual DNA strands from WT and *Dnmt3a2*$^{-/-}$ mice. 50 unique and randomly selected sequences per animal are displayed (black = methylated CpG, white = unmethylated CpG). Red box indicates the CpG site detected by the EPIC array probe.

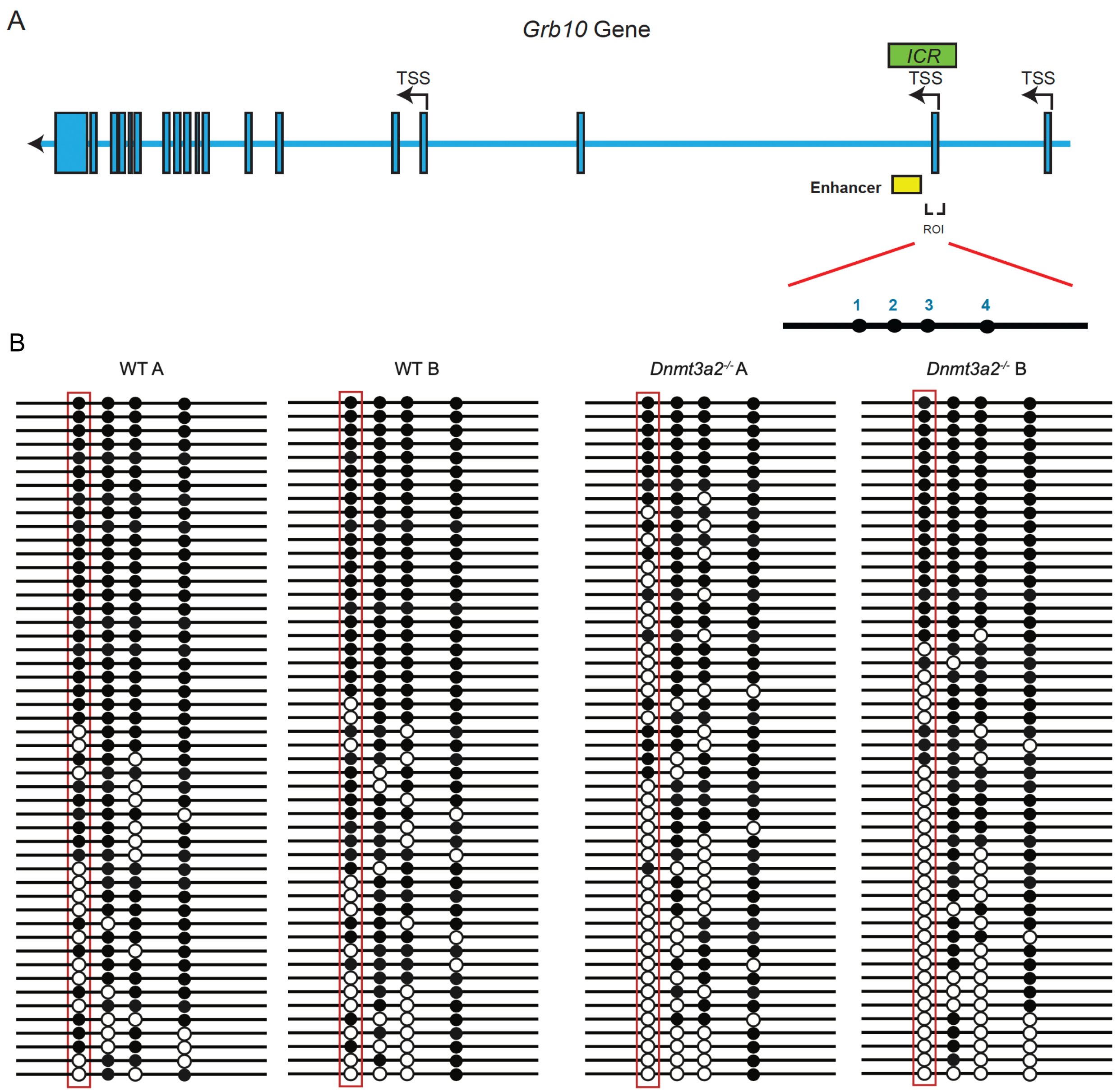

Supplementary Fig. 7: Dnmt3a2 loss induces stochastic loss of methylation at the *Grb10* gene. A. Schematic representation of the *Grb10* gene (exons are depicted as blue boxes). The region of interest (ROI) located within the ICR (green box) near an enhancer (yellow box) was amplified by targeted amplicon bisulfite sequencing to analyze the CpG dinucleotide (black bubbles) methylation. This region represents a 4 CpG-containing 211 bp amplicon. **B.** Bubble plots depicting the methylation status for *Grb10* ROI in individual DNA strands from WT and *Dnmt3a2*$^{-/-}$ mice. 50 unique and randomly selected sequences per animal are displayed (black = methylated CpG, white = unmethylated CpG). Red box indicates the CpG site detected by the EPIC array probe.

Supplementary Table 1: Oligonucleotides and primer sequences used in this study.

**gRNA sequences**	
m3a1in5g crRNA1	/AltR1/rGrArCrUrGrUrUrGrArCrGrGrArGrUrCrArCrGrGrGrUrUrUrUrAr GrArGrCrUrArUrGrCrU/AltR2/
m3a1in6g crRNA2	/AltR1/rGrCrArCrUrArGrGrArGrUrUrArArCrCrGrUrArGrGrUrUrUrUrAr GrArGrCrUrArUrGrCrU/AltR2/
mD3A2p5 crRNA1	/AltR1/rCrGrArGrUrCrCrCrCrGrGrGrUrCrArCrArArUrGrUrUrUrUrAr GrArGrCrUrArUrGrCrU/AltR2/
mD3A2p3 crRNA2	/AltR1/rUrGrGrGrArUrArGrCrCrCrArArArArCrCrGrCrGrGrUrUrUrUrAr GrArGrCrUrArUrGrCrU/AltR2/
**Genotyping Primers**	
m3a1ex5F	GGCACATGAACAAGCAAACACTAGGAG
m3a1ex5R	CCTCTCCCTCTCACAAGGAACAACC
m3a1ex6F	GATGCCAAGTGTGGAACTGGCAAAC
m3a1ex6R	CCTTGGTTCTGTCCTCAGCCATCAGAA
3A2GT5F	TGCGTGGGAGTGTGTCTATT
3A2GT5R	GCCCCAGGACAATAACTTCA
3A2GT3F	TGGACAGAGTCCTGGGGATA
3A2GT3R	CACAGGTCAACAGGAACTGC
**Primers for Bi-sulfite sequencing**	
**Region of Interest**	**Primer sequence**
H19 ICR Set1 F	GAGTATTTAGGAGGTATAAGAATT
H19 ICR Set1 R	ATCAAAAACTAACATAAACCCCT
H19 ICR Set2 F	GTAAGGAGATTATGTTTTATTTTTGG
H19 ICR Set2 R	CCTCATAAAACCCATAACTAT
H19 ROI2 F	TAGTTTGGTTTTGGTTATTTTAGTTAAT
H19 ROI2 R	CATCTAAAACTACTCTACTCTCTAATAC
Snrpn F	ATGGTGTTTTGTTTTTTTTATTTTAGAA
Snrpn R	AAATCCTAACTATCCTAAAACTCATATT
Snrpn_DMR F	ATATATTTTTGTTTTAGATTGGTATTGT
Snrpn_DMR R	TACTAATCCAACAAAAACCTAAAAAATA
Grb10 F	TAATAATTAGATTTGTAAAGGGAGGAG
Grb10 R	AAAAAATTCTATAACATAACCACCCTAA

Supplementary Table 2: dMRM parameters.

Compound name	Precursor (m/z)	Product (m/z)	RT (min)	Fragmentor (V)	CE (V)	Polarity
**2'deoxycytidine**	228	112	4.3	50	5	+
	228	95	4.3	50	40	+
**[$^{13}C_5{}^{15}N$] 2'deoxycytidine**	231	115	4.3	55	30	+
	231	157	4.3	140	15	+
	231	98	4.3	50	40	+
**2'deoxyguanosine**	268	152	7.7	25	20	+
	268	135	7.7	40	50	+
	268	110	7.7	5	40	+
**[$^{13}C_5{}^{15}N$] 2'deoxyguanosine**	271	155	7.7	30	5	+
	271	117	7.7	100	15	+
**5-methyl-2'deoxycytidine**	242	126	9.1	50	10	+
	242	108	9.1	50	40	+
**[D_3] 5-methyl-2'deoxycytidine**	245	129	9.1	50	10	+
	245	117	9.1	55	15	+
**2'deoxythymidine**	243	127	9.2	50	10	+
	243	117	9.2	50	10	+
**2'deoxyadenosine**	252	136	10.1	50	10	+
	252	119	10.1	100	50	+
	252	43	10.1	100	40	+
**[$^{13}C_5{}^{15}N$] 2'deoxythymidine**	255	134	10.65	40	2	+
	255	122	10.65	25	15	+
**2'deoxyuridine**	229	211	4.4	8	5	+
	229	113	4.4	50	20	+

Uncropped gels for western blots in figures:

Fig. 1B.

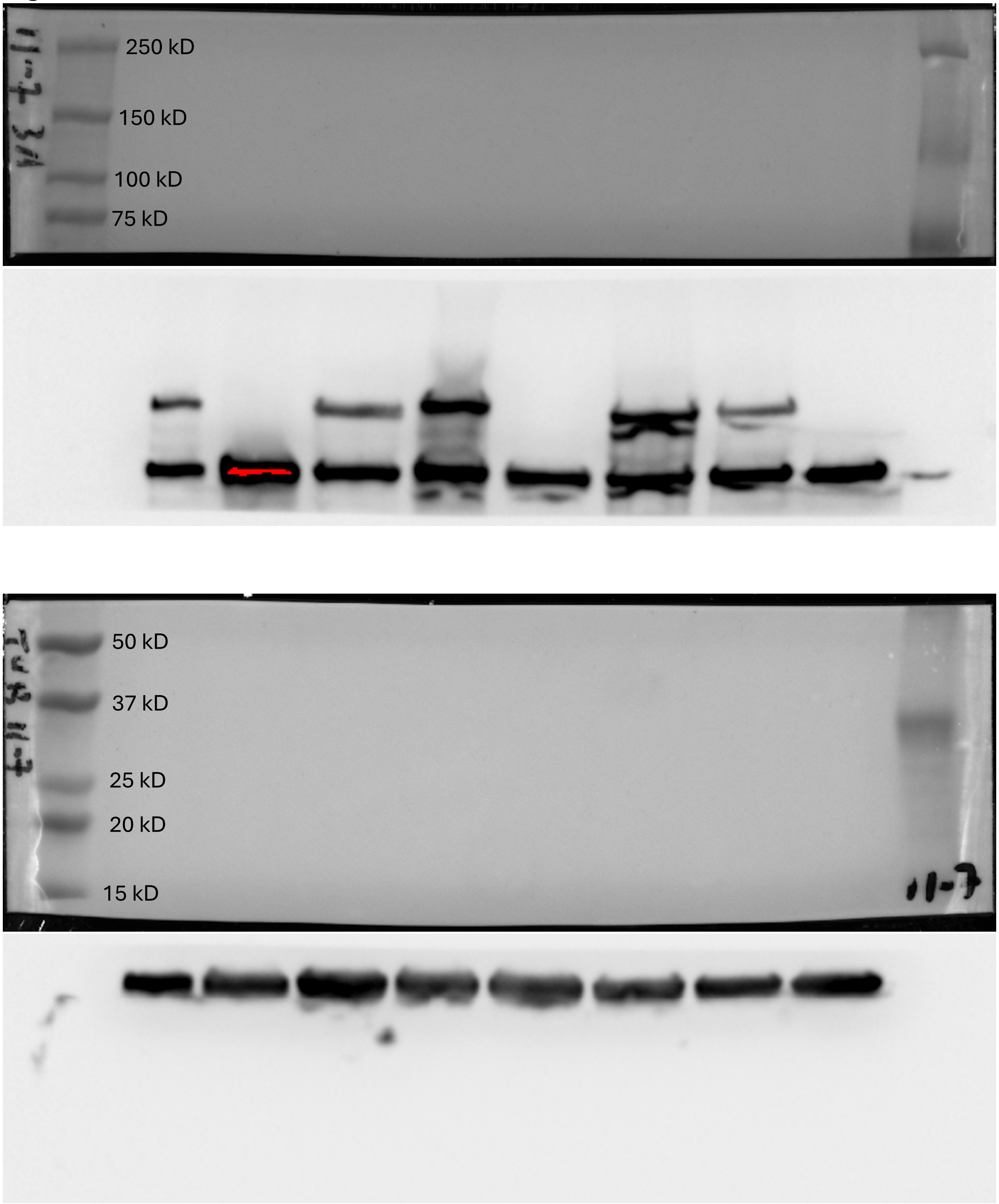

Fig. 1C.

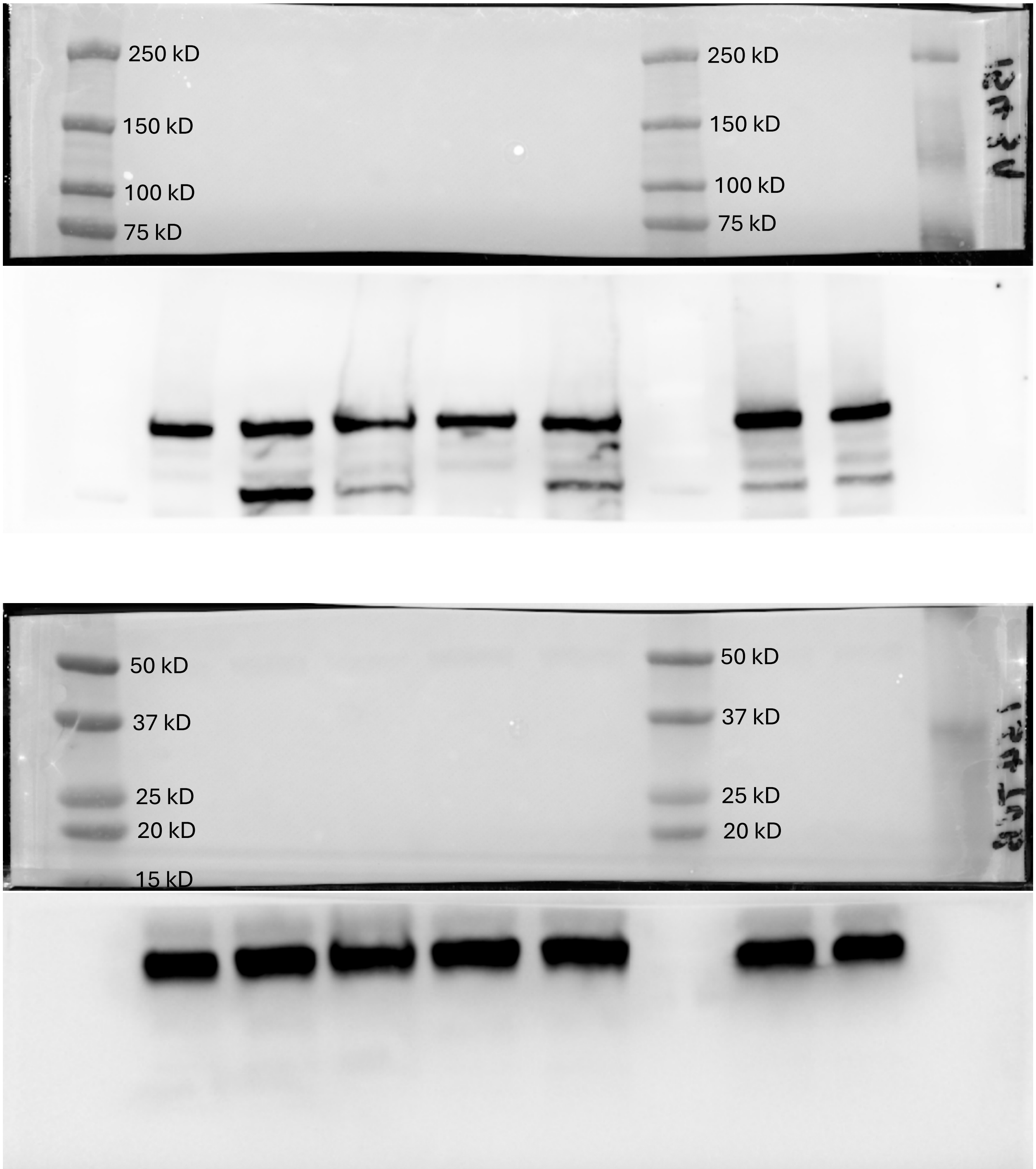
250 kD
150 kD
100 kD
75 kD
250 kD
150 kD
100 kD
75 kD
50 kD
37 kD
25 kD
20 kD
15 kD
50 kD
37 kD
25 kD
20 kD
