# Supplementary material for: Dnmt3a2 expression during embryonic development is required for phenotypic stability
Source: Commun Biol. 2025 Dec 8;9:44. doi: 10.1038/s42003-025-09311-1 (PMC12789621; doi:10.1038/s42003-025-09311-1)
Supplement: Supplementary file 3 — Description of Additional Supplementary Materials [file 42003_2025_9311_MOESM3_ESM.pdf]

Description of Additional Supplementary Files

File name: Supplementary Data

Description: All source data behind the graphs in the paper in a combined Excel file with separated tabs.
