# Supplementary material for: Dnmt3a2 expression during embryonic development is required for phenotypic stability
Source: Commun Biol. 2025 Dec 8;9:44. doi: 10.1038/s42003-025-09311-1 (PMC12789621; doi:10.1038/s42003-025-09311-1)
Supplement: Supplementary file 5 — Reporting Summary [file 42003_2025_9311_MOESM5_ESM.pdf]

nature portfolio

Corresponding author(s): Peter A. Jones

Last updated by author(s): Nov 5, 2025

Reporting Summary

Nature Portfolio wishes to improve the reproducibility of the work that we publish. This form provides structure for consistency and transparency in reporting. For further information on Nature Portfolio policies, see our Editorial Policies and the Editorial Policy Checklist.

Statistics

For all statistical analyses, confirm that the following items are present in the figure legend, table legend, main text, or Methods section.

n/a	Confirmed	
☐	☒	The exact sample size (*n*) for each experimental group/condition, given as a discrete number and unit of measurement
☐	☒	A statement on whether measurements were taken from distinct samples or whether the same sample was measured repeatedly
☐	☒	The statistical test(s) used AND whether they are one- or two-sided<br>*Only common tests should be described solely by name; describe more complex techniques in the Methods section.*
☐	☒	A description of all covariates tested
☐	☒	A description of any assumptions or corrections, such as tests of normality and adjustment for multiple comparisons
☐	☒	A full description of the statistical parameters including central tendency (e.g. means) or other basic estimates (e.g. regression coefficient) AND variation (e.g. standard deviation) or associated estimates of uncertainty (e.g. confidence intervals)
☒	☐	For null hypothesis testing, the test statistic (e.g. *F*, *t*, *r*) with confidence intervals, effect sizes, degrees of freedom and *P* value noted<br>*Give P values as exact values whenever suitable.*
☒	☐	For Bayesian analysis, information on the choice of priors and Markov chain Monte Carlo settings
☒	☐	For hierarchical and complex designs, identification of the appropriate level for tests and full reporting of outcomes
☒	☐	Estimates of effect sizes (e.g. Cohen's *d*, Pearson's *r*), indicating how they were calculated

Our web collection on statistics for biologists contains articles on many of the points above.

Software and code

Policy information about availability of computer code

Data collection	DNA methylation was assessed using Illumina Infinium Mouse Methylation BeadChip array (MM285), conducted by the VAI Genomics core (RRID:SCR_022913) following the manufacturer's specifications. Arrays were scanned on the Illumina iScan platform, and probe-specific calls were made using Illumina GenomeStudio version 2011.1 to generate IDAT files.<br>For LC/MS, Peak picking and integration was performed using Skyline Software (v 24.1).
Data analysis	Quality control, preprocessing and normalization process were performed using the SeSAMe pipeline (version 1.24.0) on Bioconductor.Probe enrichment analysis was performed using the SeSAMe knowYourCG module39, with annotations based on the knowYourCG tool, and related information available at http://zwdzwd.github.io/InfiniumAnnotation#mouse. The ChromHMM annotation was derived from a mouse consensus by the ENCODE project profiling 66 mouse epigenomes across 12 tissues at daily intervals from embryonic day 11.5 to birth40. Gene ontology analysis of probe-enriched genes was performed using the Database for Annotation, Visualization, and Integrated Discovery (DAVID) functional annotation clustering (v2023q4)41. Further data visualization of SeSAMe output was performed using RStudio (2024.12.1.563) in R (version 4.4.2).<br>For targeted bisulfite sequencing, BAM files from sorted sequences were created using SAMtools43 and loaded in IGV 2.17.4 for visualization and calculation of CpG dinucleotide methylation percentage.<br>Experimental data were analyzed using Prism 10 software (GraphPad, version 10.4.1) by one-way ANOVA, two-tailed Student's t-test or indicated in the corresponding figure legends. Statistical significance levels are denoted as follows: *,p < 0.05; **, p < 0.01; ***, p< 0.001; ****, p < 0.0001, ns, not significant. Data are presented as median ⍰ 95%CI or mean ± s.e.m. as indicated in the corresponding figure legends.<br>No statisti-cal methods were used to predetermine sample size.

For manuscripts utilizing custom algorithms or software that are central to the research but not yet described in published literature, software must be made available to editors and reviewers. We strongly encourage code deposition in a community repository (e.g. GitHub). See the Nature Portfolio guidelines for submitting code & software for further information.

Data

Policy information about availability of data

All manuscripts must include a data availability statement. This statement should provide the following information, where applicable:

- Accession codes, unique identifiers, or web links for publicly available datasets
- A description of any restrictions on data availability
- For clinical datasets or third party data, please ensure that the statement adheres to our policy

Data generated for this work have been deposited to GEO under accession number GSE295720 with the reviewer's token yfsngyyinxabdmj.

Research involving human participants, their data, or biological material

Policy information about studies with human participants or human data. See also policy information about sex, gender (identity/presentation), and sexual orientation and race, ethnicity and racism.

Reporting on sex and gender	N/A. This research did not involve human participants.
Reporting on race, ethnicity, or other socially relevant groupings	N/A. This research did not involve human participants.
Population characteristics	N/A. This research did not involve human participants.
Recruitment	N/A. This research did not involve human participants.
Ethics oversight	N/A. This research did not involve human participants.

Note that full information on the approval of the study protocol must also be provided in the manuscript.

Field-specific reporting

Please select the one below that is the best fit for your research. If you are not sure, read the appropriate sections before making your selection.

☒ Life sciences ☐ Behavioural & social sciences ☐ Ecological, evolutionary & environmental sciences

For a reference copy of the document with all sections, see nature.com/documents/nr-reporting-summary-flat.pdf

Life sciences study design

All studies must disclose on these points even when the disclosure is negative.

Sample size	No statistical methods were used to predetermine sample size.
Data exclusions	No data were excluded from analysis.
Replication	All experiments included at least two biological replicates.
Randomization	Homozygous mutant mice were generated by intercrossing heterozygous breeders. Animals from the same litter were randomly assigned to cages, with a maximum of five mice per cage. Littermate wild-type (WT) controls and mutant animals were used for all experimental comparisons to control for potential confounding effects of genetic background and environmental variables.
Blinding	Animal health and well-being were monitored daily by animal keepers and two to three times per week by trained VAI Vivarium Core staff. Both the animal keepers and Vivarium Core staff were blinded to the genotypes of individual experimental animals throughout the study to minimize potential bias in animal care and monitoring.

Reporting for specific materials, systems and methods

We require information from authors about some types of materials, experimental systems and methods used in many studies. Here, indicate whether each material, system or method listed is relevant to your study. If you are not sure if a list item applies to your research, read the appropriate section before selecting a response.

Materials & experimental systems

n/a	Involved in the study
☐	☒ Antibodies
☒	☐ Eukaryotic cell lines
☒	☐ Palaeontology and archaeology
☐	☒ Animals and other organisms
☒	☐ Clinical data
☒	☐ Dual use research of concern
☒	☐ Plants

Methods

n/a	Involved in the study
☒	☐ ChIP-seq
☒	☐ Flow cytometry
☒	☐ MRI-based neuroimaging

Antibodies

Antibodies used

DNMT3A (E9P2F) Rabbit mAb (Cell Signaling Technology CST 49768) and □-tublin (D3U1W) Mouse mAb (Cell Signaling Technology CST-86298)

Validation

The following commercially available antibodies were used in this study: DNMT3A (E9P2F) Rabbit monoclonal antibody (Cell Signaling Technology, CST 49768) and β-Tubulin (D3U1W) Mouse monoclonal antibody (Cell Signaling Technology, CST 86298). Both antibodies have been previously validated by the manufacturer for immunoblotting applications in mouse tissues and cells. Specificity was confirmed by the supplier using appropriate positive and negative controls, including DNMT3A knockdown/knockout cell lines for DNMT3A (CST 49768). In our study, expected band sizes and expression patterns consistent with the genotypes and published literature were observed, further supporting antibody specificity under the experimental conditions used.

Animals and other research organisms

Policy information about studies involving animals; ARRIVE guidelines recommended for reporting animal research, and Sex and Gender in Research

Laboratory animals

We used the following laboratory mice strains: C57BL/6 and B6C3F2.

Wild animals

N/A. This study did not involve wild animals.

Reporting on sex

Both male and female mice were included in all experiments. Phenotypic analyses were conducted on animals of both sexes, except for fertility-related assessments, where infertility was specifically observed in male mice. Data were collected from both male and female animals for all experiments, with the exception of sperm analysis, which was performed exclusively on male mice.

Field-collected samples

N/A. This study did not involve samples collected from field.

Ethics oversight

This research complies with ethical regulations, with protocols approved by the Institutional Animal Care and Use Committee (Van Andel Institute (VAI); protocols 20-06-006 and 23-06-013).

Note that full information on the approval of the study protocol must also be provided in the manuscript.

Plants

Seed stocks

N/A. This research did not involve plants.

Novel plant genotypes

N/A. This research did not involve plants.

Authentication

N/A. This research did not involve plants.
